# Feasibility of Coronary Artery Calcium Scoring on Dual-Energy Chest Computed Tomography: A Prospective Comparison with Electrocardiogram-Gated Calcium Score Computed Tomography

**DOI:** 10.3390/jcm10040653

**Published:** 2021-02-08

**Authors:** Sun Yong Lee, Tae Hoon Kim, Kyunghwa Han, Jae Min Shin, Ji Young Kim, Daein Kim, Chul Hwan Park

**Affiliations:** 1 Department of Radiology and The Research Institute of Radiological Science, Gangnam Severance Hospital, Yonsei University College of Medicine, Seoul 06273, Korea; SYLEE91@yuhs.ac (S.Y.L.); thkim1@yuhs.ac (T.H.K.); SJAEMIN@yuhs.ac (J.M.S.); flora1028@yuhs.ac (J.Y.K.); DEINA@yuhs.ac (D.K.); 2 Department of Radiology and The Research Institute of Radiological Science, Severance Hospital, Yonsei University College of Medicine, Seoul 03722, Korea; KHHAN@yuhs.ac

**Keywords:** coronary artery calcium score, correlation coefficient, dual-energy computed tomography, ECG-gated cardiac CT, wide-detector CT

## Abstract

**Rationale and Objectives**: This study aimed to evaluate the feasibility of assessment using the coronary artery calcium score (CACS) in dual-energy chest computed tomography (CT). **Materials and Methods**: We prospectively enrolled 30 patients (19 male, 11 female; mean age, 63.73 ± 9.40 years) who clinically required contrast-enhanced chest CT. The patients underwent electrocardiogram-gated cardiac calcium-scoring CT with a slice thickness of 2.5 mm followed by a sequentially non-gated contrast-enhanced dual-energy chest CT using 140/80 fast kVp switching technology with slice thicknesses of 1.25 mm and 2.5 mm. Virtual unenhanced (VUE) images were then reconstructed from the dual-energy CT using the material suppressed iodine (MSI) technique. **Results**: The mean heart rates were 63.33 ± 12.01 beats per minute. The mean CACS on the coronary calcium-scoring CT was 361.1 ± 435.5, and CACSs of the VUE images were 76.8 ± 128.6 (2.5 mm slice) and 108.7 ± 165.1 (1.25 mm slice). The correlation coefficients of CACS between the coronary calcium-scoring CT with the VUE 2.5 mm and 1.25 mm images were 0.888 and 0.904, respectively. The inter-observer agreements for the calcium score measurement between the calcium-scoring CT, VUE 2.5 mm, and VUE 1.25 mm were 1.000, 0.999, and 1.000, respectively. **Conclusions**: In conclusion, assessment of CACS using dual-energy chest CT might be feasible when using MSI virtual unenhanced dual-energy chest CT images with a slice thickness of 1.25 mm.

## 1. Introduction

Cardiovascular disease is the most common cause of death worldwide. According to the World Health Organization, 15.2 million people died from cardiovascular disease in 2016, accounting for 27% of all global deaths [1]. Computed tomography (CT) is now the modality of choice for identifying and quantitatively measuring coronary calcification using the coronary artery calcium score (CACS) [2,3]. It has been widely used to assess the clinical risk of a cardiovascular event [4,5,6]. Furthermore, CACS has been the strongest risk prediction tool among asymptomatic populations [7,8,9,10] and the most useful method to assess risk in intermediate risk populations [11,12]. To acquire CACS, electrocardiogram (ECG)-gated non-enhanced cardiac CT is needed to define coronary calcium levels using the Hounsfield unit (HU) [13].

Recently, dual-energy CT (DECT) has been widely used for chest CT examinations and two different X-ray spectra can be obtained from a single-source rapid voltage switching method or from the dual-source generating tubes [14]. Many people who need a chest CT scan may also need CACS at the same time. In the US, 6.6 million of the 7 million people who need lung scanning are expected to benefit from scanning for CACS [15]. Virtual unenhanced (VUE) images can be derived from DECT using iodine mapping and subtraction and may replace true unenhanced images, reducing radiation exposure and scan time [16,17].

Several studies evaluating the feasibility of using VUE images on DECT to acquire CACS found statistically significant correlations between them, although the CACS of VUE images was significantly lower than that of true non-enhanced cardiac CT images [18,19,20]. However, there has been no study comparing CACS using the VUE images of enhanced chest DECT with that of ECG-gated non-enhanced cardiac CT, despite the fact that ECG-gated non-enhanced cardiac CT is the reference standard for CACS. Therefore, the purpose of this study is to evaluate the feasibility of CACS evaluation using VUE images of enhanced chest DECT compared to ECG-gated non-enhanced cardiac CT.

## 2. Methods

This study had a prospective design and was approved by the Gangnam Severance Hospital ethics committee/institutional review board (3-2018-0148). Written informed consent was obtained from each patient. All methods were performed in accordance with the relevant guidelines and regulations.

### 2.1. Patient Selection

**Figure 1:**
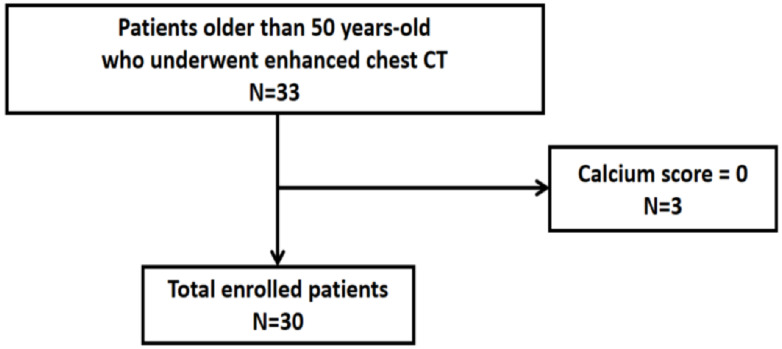
Flow chart of patient selection.

Thirty-three patients aged 50 years or older who were planned to undergo contrast-enhanced chest CT scans for any reason were prospectively enrolled in this study from August 2018 to May 2019. Exclusion criteria included age < 50 years; previous coronary artery bypass graft surgery or percutaneous coronary intervention; previous implantation with metallic devices such as pacemakers, implantable cardioverter defibrillators, or artificial heart valves; or contraindication for contrast-enhanced CT scans, such as eGFR < 60 mL/min or previous severe allergic reactions to contrast media. Three patients with a calcium score of 0 were also excluded (Figure 1).

### 2.2. CT Protocols

**Figure 2:**
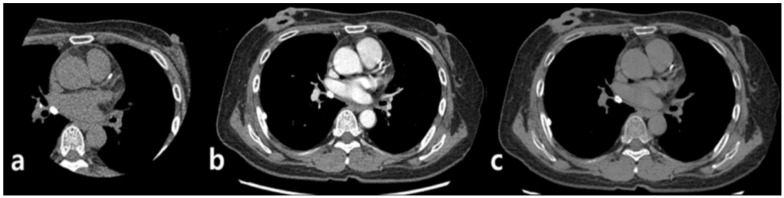
Process of image acquisition. First, ECG-gated non-enhanced calcium-scoring CT was performed (**a**). Subsequently, non-ECG-gated contrast-enhanced dual energy chest CT was performed with reconstructed virtual 70keV monochromatic images, which are similar to 120-kVp images (**b**). Finally, virtual unenhanced images were acquired with the material-suppressed iodine technique (**c**).

CT scan was performed in two steps: all patients underwent ECG-gated coronary calcium-scoring CT first and then underwent non-ECG-gated contrast-enhanced dual-energy chest CT (Figure 2).

All CT scans in this study were performed with a 256-slice CT scanner (Revolution, GE healthcare, Waukesha, Wisconsin, USA). All patients were scanned twice at the end of inspiration, in a supine position; from the aortic arch to the cardiac base by calcium score CT and from the thoracic inlet to the middle of the kidney by enhanced chest DECT.

The ECG-gated coronary calcium-scoring CT was performed with a 16cm axial volume scan and the parameters were as follows: tube voltage = 120 kVp; tube rotation time = 0.28 s; and slice thickness = 2.5 mm.

The enhanced chest DECT scans were performed using a fast kVp switching technology and an 8 cm helical scan mode with the following parameters: tube voltage = 140 kVp and 80 kVp; tube rotation time = 0.28 s; pitch = 1.531 and slice thickness = 1.25 mm. The images were acquired after a 60s administration of contrast medium (350 mg iodine = patient’s weight * 1.2 mL, limited up to 100 mL of Optiray^®^ (ioversol): Guerbet, Raleigh, NC, USA; Ultravist^®^ (Iopromide): Bayer Schering, Berlin, Germany; or Pamiray^®^ (iopamidol): Dongkook Lifescience, Seoul, Korea) and a 10s saline flush using a power injector (Nemoto Kyorindo Co., Ltd., Tokyo, Japan) (Table 1).

#### CT Image Reconstruction and Analysis

After scanning enhanced chest DECT, Gemstone Spectral Imaging data, GE’s dual-energy techniques for acquiring and generating material density data using rapid kV switching and Gemstone Detector technology, were stored in an image processing workstation (AW Server 3.2, GE Healthcare, Chicago, IL, USA) and reconstructed into 70 keV virtual monochromatic images with 1.25 mm and 2.5 mm slice thicknesses that were most similar to conventional 120 kVp polychromatic CT images [21,22,23] (Figure 2B). VUE images were then obtained using the material suppressed iodine (MSI) technique (Figure 2C).

**Figure 3:**
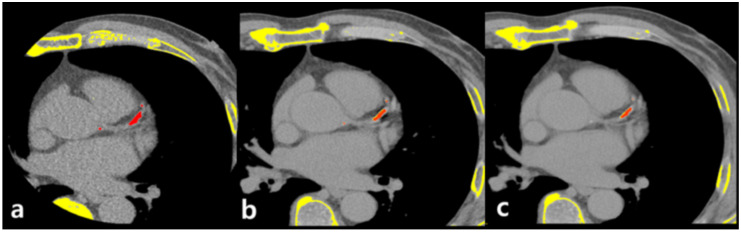
Representative case of coronary calcium scoring on calcium-scoring CT and chest dual energy CT. Pixels with HU > 130 are displayed in yellow and red if selected as calcium by operator. Calcium is well visualized on calcium-scoring CT (**a**), 1.25 mm slice thickness VUE image of chest DECT (**b**), and 2.5 mm slice thickness VUE image of chest DECT (**c**).

Two radiologists (C.H.P. and T.H.K) with >10 years of experience in chest and cardiac imaging interpretation evaluated all CT images based on consensus. CACSs were acquired with the Agatston method, using a commercially available reconstruction program for three-dimensional reconstruction and measurement (Aquarius iNtuition TM Ver.4.4.12 TeraRecon, Foster City, CA, USA). In this program, regions where the HU value is higher than 130 are displayed in yellow and are selected if they were considered as coronary calcium after reviewing slice by slice on axial images (Figure 3).

This program subsequently calculates the calcium score automatically. The Agatston scoring method, along with the multiplication of calcification areas and weights (1 = 130 to 199 HU, 2 = 200 to 299 HU, 3 = 300 to 399 HU, and 4 = 400 HU or higher), was automatically processed through the program. Images of ECG-gated non-enhanced coronary calcium-scoring CT and VUE images of chest DECT of 1.25 mm and 2.5 mm slice thickness were all analyzed.

### 2.3. Statistical Analysis

All continuous variables were expressed as mean ± standard deviation. Categorical variables were summarized as frequencies or percentages. Normality assumptions for continuous variables were tested using the Shapiro-Wilk test. A linear mixed model with Bonferroni’s method was used to evaluate the significance of differences among CACS on the coronary calcium-scoring CT and CACSs on the VUE images with 1.25mm and 2.5mm slice thicknesses. Spearman’s correlation analysis was used to evaluate correlations between CACS on the coronary calcium-scoring CT and CACSs on the VUE images. Bland-Altman analysis was used to evaluate the limits of agreement between CACS on the coronary calcium-scoring CT and CACSs on the VUE images. For the sectional analysis, CACSs were divided into ranges of 0, 0–10, 10–100, 100–400, and ˃400 for risk classification [6]. Then, the VUE images of enhanced chest DECT (1.25 mm and 2.5 mm) were compared with those of the coronary calcium-scoring CT in all patients. After linear regression analysis, CACS of the coronary calcium-scoring CT was estimated using that of the VUE images of enhanced chest DECT and regression equations, and compared with those of coronary calcium-scoring CT. Cohen’s kappa coefficient was used to analyze agreement for sectional analysis between VUE images of enhanced chest DECT and coronary calcium-scoring CT for CACS. Inter-observer reproducibility in measuring the calcium score was evaluated with the ICC. A *p*-value of less than 0.05 was considered statistically significant. All statistical analyses were performed with IBM SPSS Statistics for Windows, version 25.0 (IBM Corp., Armonk, N.Y., USA) or MedCalc Version 19.6.4 (MedCalc Software bv, Ostend, Belgium; https://www.medcalc.org; 2021)

## 3. Results

### 3.1. General Characteristics of the Patients

In total, 33 patients were enrolled in our study; however, three patients were excluded because their CACS was 0. The mean age of the remaining 30 patients was 63.73 ± 9.40 years, with a male:female ratio of 19:11. Most of the patients (27/30) underwent CT scans for cancer evaluation, two of which were lung cancers, and three patients underwent CT scans because of abnormal chest radiographs. The mean height of the patients was 163.17 ± 7.45 cm; mean weight was 63.93 ± 9.64 kg; and mean body mass index was 24.00 ± 3.16 kg/m^2^. The mean heart rate was 63.33±12.01 beats per minute. Among the 30 patients, five (16.7%) had diabetes mellitus, seven (23.3%) had hypertension, five (16.7%) had hyperlipidemia, and three (10.0%) were current smokers. There was no patient who had a history of ischemic heart disease or had a previous myocardial infarction.

The mean radiation exposure from chest DECT was 276.74 ± 57.30 mGy*cm, whereas the mean radiation exposure from calcium-scoring CT was 26.61 ± 10.33 mGy*cm (Table 2).

### 3.2. Comparison of CACS between Coronary Calcium-Scoring CT and VUE Images from Chest DECT

The mean CACS on the coronary calcium-scoring CT was 361.1 ± 435.5. The mean CACSs on the VUE images with 1.25 mm and 2.5 mm slice thicknesses were 108.7 ± 165.1 and 76.8 ± 128.6, respectively (Table 3). The mean CACS of each slice thickness was significantly lower than that of corresponding coronary calcium-scoring CT (*p* < 0.001, each).

There were one and eight cases of false negative CACS on VUE images with 1.25 mm and 2.5 mm slice thicknesses, respectively.

**Figure 4:**
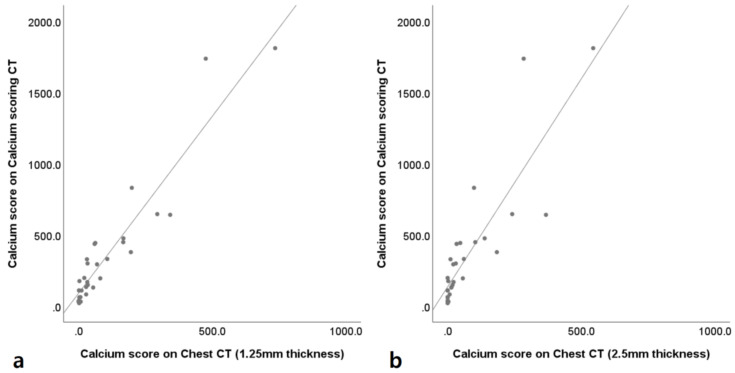
Correlation between calcium-scoring CT and chest dual energy CT. Scatter plots between calcium-scoring CT and virtual unenhanced (VUE) images of enhanced chest dual energy CT (DECT) with 1.25 mm slice thickness (**a**) and 2.5 mm slice thickness (**b**). The correlation coefficients of coronary artery calcium scoring between the coronary calcium-scoring CT and VUE images of chest DECT with 1.25 mm and 2.5 mm slice thicknesses are 0.904 and 0.888.

The correlation coefficients of CACS between the coronary calcium-scoring CT and VUE images of chest DECT with 1.25 mm and 2.5 mm slice thicknesses were 0.904 and 0.888, respectively (Figure 4).

**Figure 5:**
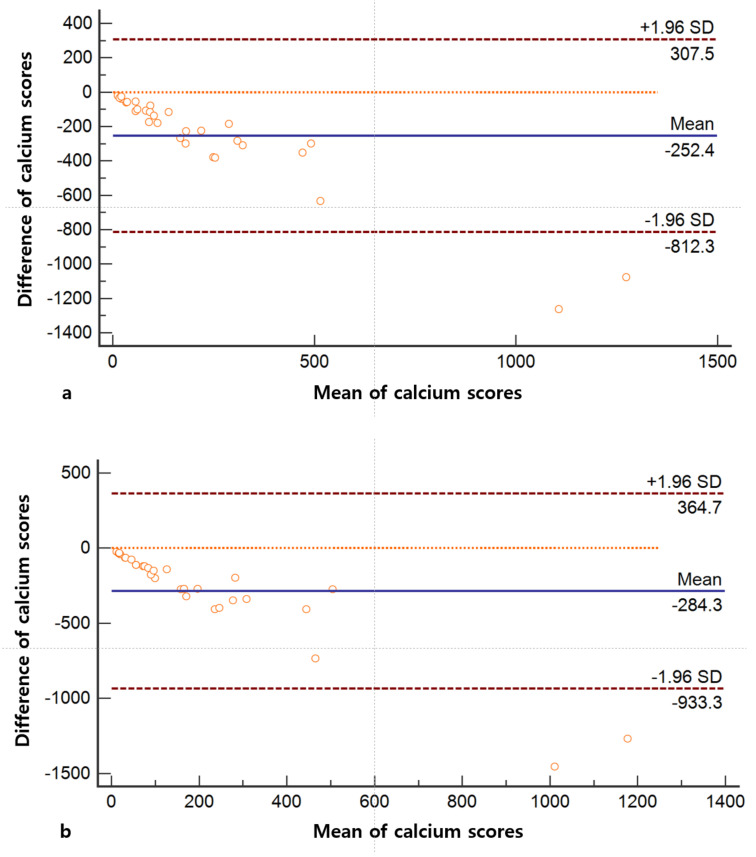
Bland-Altman plots of the coronary calcium-scoring CT and chest dual energy CT. Bland-Altman plots between the coronary calcium-scoring CT and chest dual energy CT with 1.25 mm slice thickness (**a**) and 2.5 mm slice thickness (**b**).

The mean differences of CACS between the coronary calcium-scoring CT and VUE images of chest DECT with 1.25 mm and 2.5 mm slice thicknesses were −252.4 (95% limit of agreement: −812.3 and 307.5) and −284.3 (95% limit of agreement: −933.3 and 364.7) (Figure 5).

### 3.3. Sectional Analysis of CACS

After dividing patients’ CACS values into ranges of 0, 0–10, 10–100, 100–400, and ˃400, incidences of underestimation of CACSs of VUE images were easily detected, with this occurring more frequently in 2.5 mm slice thickness images than in the 1.2 5mm slice thickness images. Only five patients of 30 in the 1.25 mm slice thickness images and two patients of 30 in the 2.5 mm slice thickness images were classified into the same section of the calcium score; the proportions of agreement were 16.67% and 6.67%, respectively. Cohen’s kappa coefficients of sectional analysis were 0 for VUE images with 1.25 mm and 0 for VUE images with 2.5 mm (Table 4 and Table 5).

After estimation of CACSs on coronary calcium-scoring CT from VUE images of chest DECT using first order linear regression analysis, the proportion of agreement was 73.33% (22/30) for VUE images with 1.25-mm slice thickness and 66.67% (20/30) for VUE images with 2.5-mm slice thickness. Cohen’s kappa coefficients of VUE images with 1.25-mm and 2.5-mm were 0.573 and 0.423, respectively (Table 6 and Table 7).

### 3.4. Inter-Observer Agreement

The inter-observer agreements for the CACS measurement between the calcium-scoring CT, VUE images with 1.25 mm slice thickness, and VUE images with 2.5 mm slice thickness were essentially perfect, at 1.000, 1.000, and 0.992, respectively.

## 4. Discussion

Our prospective study shows that CACSs of VUE images from single-source fast kVp-switching enhanced chest DECT correlates closely with those of the ECG-gated non-enhanced coronary calcium-scoring CT. Correlation coefficients of CACSs between the coronary calcium-scoring CT with VUE images of chest DECT were very high, especially for 1.25 mm slice thickness images.

Coronary artery disease is a major cause of mortality globally, and atherosclerotic changes in the coronary artery constitute the main pathophysiology of CAD [13,24]. CACS may represent an atherosclerotic burden and independently predict coronary events [8]. Conventionally, CACS should be based on ECG-gated non-enhanced calcium score CT with predefined parameters [25]. However, it is possible to calculate CACS based on chest CT images. In 2016, the Society of Cardiovascular Computed Tomography (SCCT) and the Society of Thoracic Radiology (STR) recommended coronary artery calcium scoring based on non-enhanced chest CT images in a jointly published guideline [13]. Many studies have been conducted on CACS acquisition without ECG-gated non-enhanced coronary calcium-scoring CT. Some studies showed a correlation between CACS from non-ECG-gated chest CT [24,25], or even non-ECG-gated low-dose chest CT [26,27], and CACS from ECG-gated non-enhanced coronary calcium-scoring CT. Several other studies have tried to assess the feasibility of CACS from VUE images of DECT. Song et al. [19] compared 54 patients’ VUE images from single-source fast switching enhanced dual-energy chest CT (140 kVP and 80 kVP) with non ECG-gated non-contrast chest CT (120 kVP). They acquired VUE images from not only the MSI, but also material density iodine-water pair and material density iodine-calcium pair images, and all showed excellent correlation. CACS values of MSI images were approximately 1/3 to 1/4 of those from the calcium score CT after linear regression. Yamada et al. [20] compared 27 patients’ VUE images from single-source fast switching enhanced dual-energy cardiac CT (70 kVP and 140 kVP) with ECG-gated non-contrast coronary calcium-scoring CT. CACSs of VUE images were about 1/2 of those from coronary calcium-scoring CT after linear regression; the stronger correlation was probably due to the thin slice thickness used (0.625mm). Schwarz et al. [18] compared 36 patients’ VUE images from dual-source enhanced dual-energy cardiac CT (100 kVP and 140 kVP) with ECG-gated non-contrast coronary calcium-scoring CT. They compared the calcium volume rather than the Agatston calcium score and found an excellent correlation.

VUE images of chest DECT showed some false negative results: one case in the 1.25 mm slice thickness images and eight cases in the 2.5 mm slice thickness images. We reviewed the eight false negative cases from the 2.5 mm VUE images. All had several to multiple tiny calcified plaques; seven cases had calcified plaques in the left anterior descending artery, four cases in the right coronary artery, and two cases in the left circumflex artery. The minimum heart rate was 57 beats per minute and the maximum heart rate was 80 beats per minute without definite motion artifact. One case also showed a false negative result on a 1.25 mm slice thickness image. In seven of eight patients, CACSs were less than 100 on coronary calcium-scoring CT, and one patient’s CACS was more than 100 with long segmental thin calcifications in the left anterior descending artery and right coronary artery. Multiple regions selected as calcium by coronary calcium-scoring CT also showed high attenuation on 2.5 mm VUE examination, but their attenuations were less than 130 HU and were not selected as calcium.

According to our results, the CACS of VUE images from enhanced chest DECT is significantly lower than obtained by routine coronary calcium-scoring CT. This is consistent with previous studies that used a three-material decomposition algorithm [18,20] and two-material decomposition algorithm [19]. This result may be due to artefacts related to cardiac movement with non-ECG-gated images of VUE images, blooming or beam hardening from 80 kVP images of DECT [23,28], and erroneous subtraction of calcium in post-processing since the attenuation/keV curve of 6% iodine is similar to that of calcium. Furthermore, VUE images with 2.5 mm slice thickness had more underestimated CACSs than VUE images with 1.25 mm slice thickness; this may be due to a partial volume artefact, which results in lowering of the HU of true calcium levels. These underestimations may have been amplified because the Agatston method uses a weighted density score based on high HU. However, CACS can be calculated for patients who undergo chest DECT, given the high correlation between CACS based on VUE images and CACS based on coronary calcium scoring CT. Furthermore, automatic CACS may accelerate the clinical use of CACS based on chest CT images [29].

Our study had several limitations. First, the number of patients of our study was small and included a relevant number of false negatives. Second, the CACSs of VUE images were significantly lower than those of coronary calcium-scoring CT, and there were several false negative results. However, the CACS of the false negative result in the 1.25 mm VUE image was less than 100 (29.47), and this may be a low score with low CVD risk. Lastly, there is no external validation test for the first-order linear regression equation. Further studies are needed with a larger sample size.

In conclusion, the assessment of CACS using dual-energy chest CT shows excellent correlation with CACS from ECG-gated coronary calcium-scoring CT. It might be feasible to evaluate CACS using the MSI virtual unenhanced images from enhanced dual-energy chest CT images with 1.25 mm slice thickness. However, underestimation and false negatives on the CACS of VUE should be considered, and further studies are needed with a larger sample size.

## Figures and Tables

**Table 1:** Protocols for calcium-scoring CT and chest DECT.

	Calcium-Scoring CT	Dual-Energy Enhanced Chest CT
Tube voltage (kVp)	120	80/140 fast switching
Tube currents (mAs)	50	AEC
Slice thickness (mm)	2.5	1.25, 2.5
Coverage length (cm)	16	8
Scan mode	Axial	Helical
Rotation time(sec)	0.28	0.28
ECG gating	Done	None
Contrast media	Not used	Used

AEC, automatic exposure control; CT, computed tomography; DECT, dual-energy computed tomography; ECG, electrocardiogram.

**Table 2:** Demographic data and baseline characteristics of the 30 patients enrolled.

N = 30	
**Age (years-old)**	63.73 ± 9.40
**Gender (M:F)**	19:11
**Height (cm)**	163.17 ± 7.45
**Weight (kg)**	63.93 ± 9.64
**BMI (kg/m^2^)**	24.00 ± 3.16
**Heart rate (beats/min)**	63.33 ± 12.01
Radiation exposure (mGy*cm)	Calcium CT: 26.61 ± 10.33
	Chest DECT: 276.74 ± 57.30

BMI, body mass index; CT, computed tomography; DECT, dual-energy computed tomography; F, female; M, male.

**Table 3:** Coronary artery calcium scores of calcium scoring CT and VUE images using chest DECT.

	1.25 mm	2.5 mm
**Calcium scoring CT**		361.1 ± 435.5
**VUE images of chest DECT**	108.7 ± 165.1	76.8 ± 128.6

CT, computed tomography; DECT, dual-energy computed tomography; VUE, virtual un-enhanced.

**Table 4:** Sectional analysis of CACS of CT and VUE images using chest DECT with 1.25 mm slice thickness.

	VUE Images of Chest DECT 1.25 mm					
**Calcium scoring CT 2.5 mm**		**0**	**<10**	**10~<100**	**100~<400**	**400~**
	**0**					
	**<10**					
	**10~<100**	1	5	**1**		
	**100~<400**		2	10	**2**	
	**400~**			2	5	**2**

CT, computed tomography; DECT, dual-energy computed tomography; VUE, virtual un-enhanced; CACS, coronary artery calcium scoring.

**Table 5:** Sectional analysis of CACS of CT and VUE images using chest DECT with 2.5 mm slice thickness.

	VUE Images of Chest DECT 2.5 mm					
**CALCIUM scoring CT 2.5 mm**		**0**	**<10**	**10~<100**	**100~<400**	**400~**
	**0**					
	**<10**					
	**10~<100**	5	2			
	**100~<400**	3	1	9	**1**	
	**400~**			3	5	**1**

CT, computed tomography; DECT, dual-energy computed tomography; VUE, virtual un-enhanced; CACS, coronary artery calcium scoring.

**Table 6:** Sectional analysis of CACS of CT and estimated CACS from VUE images using chest DECT with 1.25 mm slice thickness and the first-order linear regression equation.

	Estimated CACS from VUE Images Using Chest DECT 1.25 mm					
**Calcium scoring CT 2.5 mm**		**0**	**<10**	**10~<100**	**100~<400**	**400~**
	**0**					
	**<10**					
	**10~<100**			**4**	2	
	**100~<400**			3	**11**	2
	**400~**				1	**7**

CT, computed tomography; DECT, dual-energy computed tomography; VUE, virtual un-enhanced; CACS, coronary artery calcium scoring.

**Table 7:** Sectional analysis of CACS of CT and estimated CACS from VUE images using chest DECT with 2.5 mm slice thickness and the first-order linear regression equation.

	Estimated CACS from VUE Images Using Chest DECT 2.5 mm					
**Calcium scoring CT 2.5 mm**		**0**	**<10**	**10~<100**	**100~<400**	**400~**
	**0**					
	**<10**					
	**10~<100**			**0**	0	
	**100~<400**			7	**13**	2
	**400~**			0	1	**7**

CT, computed tomography; DECT, dual-energy computed tomography; VUE, virtual un-enhanced; CACS, coronary artery calcium scoring.

## Data Availability

The data presented in this study is provided upon request of the corresponding author. Data cannot be used publicly due to privacy protection.

## Abbreviations

CACS	Coronary artery calcium score
CT	Computed tomography
DECT	Dual-energy computed tomography
ECG	Electrocardiogram
MSI	Material suppressed iodine
VUE	Virtual unenhanced
